# Extended-spectrum β-lactamases producing multidrug resistance *Escherichia coli*, *Salmonella* and *Klebsiella pneumoniae* in pig population of Assam and Meghalaya, India

**DOI:** 10.14202/vetworld.2018.868-873

**Published:** 2018-06-29

**Authors:** A. Lalruatdiki, T. K. Dutta, P. Roychoudhury, P. K. Subudhi

**Affiliations:** Department of Veterinary Microbiology, College of Veterinary Science and A.H., Central Agricultural University, Selesih, Aizawl - 796 014, Mizoram, India

**Keywords:** *Enterobacteriaceae*, multidrug resistance, North East India, pigs

## Abstract

**Aim:**

The present study was conducted to record the prevalence of extended spectrum β-lactamases (ESBLs) producing *Escherichia coli*, *Salmonella* spp., and *Klebsiella pneumoniae* from pig population of Assam and Meghalaya and to record the ability of the resistant bacteria to transfer the resistance genes horizontally.

**Materials and Methods:**

Fecal samples (n=228), collected from pigs of Assam (n=99) and Meghalaya (n=129), were processed for isolation and identification of *E. coli* and *Salmonella* spp. All the isolates were tested for ESBLs production by double disc synergy test (DDST) followed by screening for ESBLs producing genes (*bla*_TEM_, *bla*_SHV_, *bla*_CTX-M_, and *bla*_CMY_) by polymerase chain reaction (PCR). Possible transfer of resistance encoding genes between enteric bacterial species was carried out by *in vitro* and *in vivo* horizontal gene transfer (HGT) method.

**Results:**

A total of 897 enteric bacteria (867 *E. coli* and 30 *Salmonella*) were isolated and identified. Altogether 25.41% isolates were confirmed as ESBL producers by DDST method. Majority of the isolates were *E. coli* followed by *Salmonella*. By PCR, 9.03% isolates were found positive for at least one of the target resistance genes. *bla*_SHV_ was absent in all the isolates. *bla*_CMY_ was the most prevalent gene. All the *E. coli* isolates from Assam were negative for *bla*_TEM_. A total of 2.76% isolates were positive for *bla*_TEM_ + *bla*_CMY_. On the other hand, 0.67% isolates were positive for *bla*_CTX-M_ + *bla*_CMY_ genes. Only 0.33% isolates carried all the three genes. Altogether, 4.68% bacteria carried the resistance encoding genes in their plasmids. *bla*_TEM_ gene could be successfully transferred from *Salmonella* (donor) to *E. coli* (recipient) by *in vitro* (5.5-5.7×10^−5^) and *in vivo* (6.5×10^−5^ to 8.8×10^−4^) methods. *In vivo* method was more effective than *in vitro* in the transfer of resistance genes.

**Conclusion:**

The pig population of Assam and Meghalaya are carrying multidrug resistance and ESBLs producing *E. coli* and *Salmonella*. The isolates are also capable to transfer their resistance trait to other bacterial species by HGT. The present finding could be considered as a serious public health concern as similar trait can also be transmitted to the human commensal bacteria as well as pathogens.

## Introduction

In large-scale poultry and livestock production facilities, antimicrobial agents may be used for therapeutic, prophylactic, and/or sub-therapeutic growth promotion purposes. Such use of antimicrobial agents puts selective pressure on commensals and pathogenic bacteria contributing to the clonal expansion of multidrug-resistant (MDR) bacteria [1]. The use of antibiotics in farm animals that are critically important in human medicine is implicated in the emergence of new forms of MDR bacteria particularly the bacterial species under family Enterobacteriaceae. The problem of MDR is a major public health concern due to its global dimension and alarming magnitude, although the epidemiology of resistance can exhibit a remarkable geographical variability and a rapid temporal evolution [2]. The rapid emergence of extended spectrum β-lactamases (ESBLs) made a serious impact on the treatment and therapeutic strategy of clinical infections [3] mainly caused by the enteric bacteria, namely *Escherichia coli*, *Salmonella* spp., and *Klebsiella* *pneumoniae*.

Food animals, including pigs, are one of the most important sources of development of MDR bacteria because of continuous use of antibiotics as feed additives and growth promoting factors at a sub-therapeutic level [4]. This practice may lead to the selection of a resistant population in the native microbiota of the animal and the local environment due to shedding through feces. The MDR bacteria may re-enter the human and animal populations through various routes including natural water, irrigation water, drinking water, vegetables, and foods.

Although a handful of sporadic report on MDR enteric bacteria of animal origin from different parts of India is available, so far, very little is known about the MDR bacteria in pigs of Northeastern region of India [5]. The present study was undertaken to record the prevalence of ESBLs producing *E. coli*, *Salmonella* spp., and *K. pneumoniae* from pig population of Assam and Meghalaya and to record the ability of the resistant bacteria to transfer the resistance genes horizontally.

## Materials and Methods

### Ethical approval

The animal experimentation was approved by The Institutional Animal Ethics Committee, College of Veterinary Science & A.H., CAU, Aizawl, Mizoram and all samples were collected by skilled personnel without any harm to the animals.

### Sampling and isolation of bacteria

A total of 228 fecal samples of pigs from Assam (n=99) and Meghalaya (n=129) were collected irrespective of their sex, age, or breed. All the samples were processed for isolation and identification of *E. coli*, *Salmonella* spp., and *K. pneumoniae* by standard bacteriological techniques.

### Antimicrobial sensitivity and detection of ESBLs production by the phenotypic method

All the isolates were processed for antimicrobial susceptibility test on Mueller-Hinton agar plate as per the recommendation of Clinical Laboratory Standard Institute (CLSI) [6] using commercially available antibiotic disks, namely ceftriaxone, ceftazidime, cefixime, cefotaxime, ampicillin, cefalexin, gentamicin, ciprofloxacin, piperacillin, amoxicillin, imipenem, aztreonam, streptomycin, and nalidixic acid. Isolates showing resistance to the third generation cephalosporins were subjected to double disc synergy test (DDST) by placing cefotaxime and cefotaxime/clavulanate, amoxicillin and amoxicillin/clavulanate, and ceftazidime and ceftazidime/clavulanate disks in each plate of Mueller-Hinton agar at a distance of 25 mm apart as per the recommendation of CLSI [6]. Plates were incubated overnight, and the increase in zone size more than 5 mm was considered as positive for ESBLs production.

### Extraction of plasmid and chromosomal DNA

Plasmid DNA and chromosomal DNA were extracted from all the isolates found to be positive for ESBLs using modified alkaline lysis method.

### Polymerase chain reaction (PCR) detection of selected ESBLs producing genes

All the suspected ESBLs producing isolates were subjected to PCR for detection of ESBLs producing genes (*bla*_TEM_, *bla*_SHV_, *bla*_CTX-M_, and *bla*_CMY_) using specific primers [5]. PCR was carried out in a 0.2 ml thin wall PCR tubes with a final volume of 25 µl containing 10× buffer, 1.5 mM MgCl_2_, 200 pM of each oligonucleotide primers, 200 µM of each dNTPs, 1 U of Taq polymerase, and 50 pg DNA. The reaction was carried out in a thermal cycler (Mastercycler Nexus Gradient, Eppendorf, Germany) using the following temperature regimes: Initial denaturation at 94°C for 5 min followed by 30 cycles of amplification with denaturation at 94°C for 30 s (SHV and TEM), 94°C for 45 s (CMY and CTX-M); annealing at 58°C for 30 s (SHV and TEM), 60°C for 45 s (CMY), and 53°C for 45 s (CTX-M); and extension at 72°C for 1 min with final extension at 72°C for 6 min. PCR was carried out for all the four genes separately using genomic DNA and plasmid DNA to locate the target genes.

### Curing of plasmid

All the isolates carrying selected ESBLs genes in their plasmid were subjected to curing using acridine orange as per the method described by Silhavy *et al*. [7]. In brief, 0.2 ml of overnight culture was added in 5 ml LB broth containing different concentrations and incubated overnight at 37°C in shaking incubator. Positive control contained only cells with no acridine orange, while negative control contained only acridine orange without cells in LB broth. Next day, the tubes containing the highest concentration of acridine orange showing growth were selected, and loopful of the culture was streaked on MLA plate and incubated at 37°C overnight. Colonies from the plates were then checked for the loss of antibiotic resistance by disc diffusion method.

### Selection of donor and recipient organism

*Salmonella* (DS-54) isolates with the antibiotic resistance pattern of amoxicillin, aztreonam, cefixime, cefotaxime, ceftazidime, ceftriaxone, cephalexin, and streptomycin was studied for the presence of target resistance genes (*bla*_TEM_, *bla*_CTX-M_, and *bla*_CMY_). The isolate was found to be positive for *bla*_TEM_ and *bla*_CMY_ genes in its plasmids were selected as donor strain. *E. coli* (DEC-17) was used as recipient strain, which was sensitive to nalidixic acid, ceftazidime, ceftriaxone, cefotaxime, and streptomycin and was also negative for *bla*_TEM_ and *bla*_CMY_ genes in their plasmid as confirmed by PCR analysis.

### *In vitro* horizontal gene transfer (HGT)

*In* *vitro* HGT was performed by broth mating, filter paper mating, and plate mating methods. In broth mating [8] cultures of recipient and donor strains were inoculated separately in LB broth and incubated overnight at 37°C and a 1:2 ratio (donor to recipient) of the culture was inoculated into fresh LB broth and incubated for 3 h at 37°C in a shaking incubator. Samples (0.1 ml) of this mixture were spread onto the surface of Hektoen enteric agar (HEA) plates containing streptomycin. In filter paper mating donor and recipient bacteria were grown in LB broth to logarithmic phase at 37°C and a 1:4 ratio (v/v) of the donor and recipient culture were collected on a sterile filter paper. The filter paper containing both the bacteria placed on HEA and incubated at 37°C for 20 h. In case of plate mating, the donor and recipient isolates were grown separately on HEA plates containing streptomycin and incubated overnight at 37°C. Colonies appearing in the cross area were pooled and spread on separate HEA containing streptomycin and cefixime. Donor and recipient strains were grown separately in antibiotic-free medium as well as in antibiotic medium as a control. *E. coli* colonies grown on the selection plates were subjected to DDST to confirm the presence of ESBL transconjugants. The frequency of transfer was expressed relative to the number of donor cells. Selected transconjugants (*E. coli*) were further characterized for their antimicrobial susceptibility, ESBL phenotype, plasmid profiling, and the presence of *bla*_TEM_ and *bla*_CMY_ genes by PCR.

### *In vivo* mating

*In vivo* mating experiment was performed using mouse model. The mating experiment was performed with and without any antibiotic pressure. 6-8 weeks old, outbred Swiss albino mice were used for colonization and antimicrobial resistance transfer studies. A total of 6 mice were used for the experiment. Mouse numbers M1, M2, and M3 were grouped under antibiotic treatment, whereas mouse numbers M4, M5, and M6 were grouped under non-antibiotic treatment. The mice were individually caged, and cages were changed daily. The mice had unlimited access to food and continuously received either pure drinking water (M4, M5, and M6) or water containing streptomycin sulfate (0.5 g/L) (M1, M2, and M3) throughout the study. Before inoculation of the donor or recipient strains, fecal samples from each mouse were tested for the presence of indigenous bacteria with similar resistance. All the six mice were fed with the recipient strain *E. coli* (DEC-17) (2×10^9^ cfu/mouse) *per os*. The numbers of c.f.u. in fecal samples were determined on HEA plates containing 14 mg/ml streptomycin. On the 7^th^ day, the donor strain *Salmonella* sp. (DS-54) was fed at 2×10^9^ cfu/mouse *per os*. Fecal samples were collected from each mouse on days 2, 4, 6, 8, and 10 postinoculation. The samples were processed for detection of donor, recipient, and transconjugants on selective medium. Frequency of the transfer of resistance genes from donor to recipient strains was determined by the ratio of transconjugants and donor. Selected transconjugants (*E. coli*) were further characterized for their antimicrobial susceptibility, ESBL phenotype, plasmid profiling, and the presence of *bla*_TEM_ and *bla*_CMY_ genes by PCR.

### Determination of minimal inhibitory concentration (MIC) by agar plate dilution method

MIC was determined only to those isolates, which were used for conjugation study. Isolate number DS-54 was used as donor and isolate number DEC-17 was used as a recipient for determining their MIC using streptomycin.

## Results

### Bacterial isolates

A total of 897 enteric bacteria (867 *E. coli* and 30 *Salmonella*) were isolated from 228 fecal samples of pigs. Of the 867 (96.65%) *E. coli* isolates, 537 were from Meghalaya, and 330 were from Assam. Similarly, of the 30 (3.34%) *Salmonella* spp. isolates, 24 were from Meghalaya and 6 were from Assam. *K. pneumoniae* could not be isolated from any samples under the study.

### Phenotypic detection of ESBLs production

Based on the observation of antimicrobial sensitivity assay, a total of 450 (50.16%) isolates were found to be positive and suspected for ESBL production, of which 429 (49.48 %) were *E. coli* (216 from Meghalaya and 213 from Assam) and 21 (70.00 %) were *Salmonella* spp. (15 from Meghalaya and 6 from Assam). Of the 450 isolates, 228 (25.41%) were confirmed as ESBL producers based on the DDST screening method. Of the 228 ESBLs producing isolates, 210 (24.22%) and 18 (60.00%) were *E. coli* and *Salmonella* spp., respectively.

### Genotypic characterization of ESBLs production

**Figure-1:**
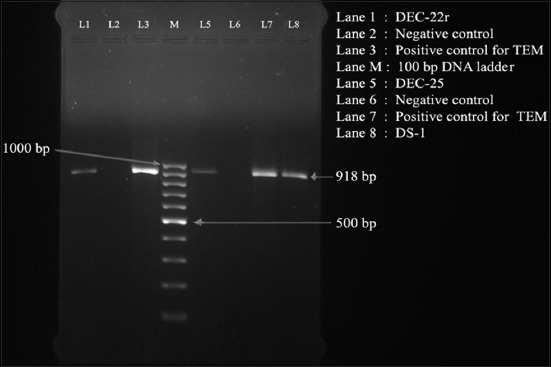
Polymerase chain reaction assay for detection of *bla*_TEM_ gene (918 bp) in *Escherichia coli* isolated from pigs of NER starts of India. Lane 1: DEC-22; Lane 2: Negative control; Lane 3: Positive control for TEM; Lane M: 100 bp DNA ladder; Lane 5: DEC-25; Lane 6: Negative control; Lane 7: Positive control for TEM; and Lane 8: DS-1.

**Figure-2:**
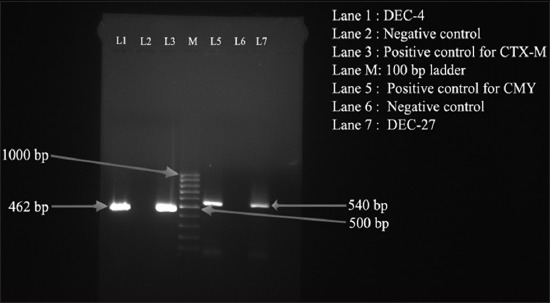
Polymerase chain reaction-based detection of *bla*_CTX-M_ (462 bp) and *bla*_CMY_ (540 bp) genes *Escherichia coli* isolated from pigs of NER starts of India. Lane 1: DEC-4; Lane 2: Negative control; Lane 3: Positive control for CTX-M; Lane M: 100 bp DNA ladder; Lane 5: Positive control for CMY; Lane 6: Negative control; and Lane 7: DEC-27.

Of the 210 ESBL producing *E. coli* isolates confirmed by DDST method, 66 (7.61%) were found to be positive for at least one of the three ESBLs gene, namely *bla*_TEM_ (918 bp), *bla*_CTX-M_ (540 bp), and *bla*_CMY_ (462 bp) (Table-1, Figures-1 and 2). Of the 66 isolates, 15 (1.73%), 9 (1.03%), and 24 (2.76%) were found to be positive for *bla*_TEM_, *bla*_CTX-M_, and *bla*_CMY_, respectively (Table-1). A total of 15 (1.73%) and 3 (0.90%) isolates were positive for combination of *bla*_TEM_ + *bla*_CMY_ and *bla*_CTX-M_ + *bla*_CMY_ genes, respectively. Similarly, of the 21 *Salmonella* isolates, 12 were positive for at least one of the three ESBLs gene (Table-1), of which 3 (10.00%) isolates were positive for *bla*_CMY_ gene. A total of 6 (20.00%) and 3 (10.00%) isolates were positive for combination of *bla*_TEM_ + *bla*_CMY_ and *bla*_CTX-M_ + *bla*_TEM_ + *bla*_CMY_ genes, respectively.

**Table-1:** Distribution of *bla* genes (*bla*_TEM_, *bla*_CTX-M_, and *bla*_CMY_) in *E. coli* and *Salmonella* spp. Isolated form pigs in Meghalaya and Assam.

Genes	*E. coli* (%)			*Salmonella* (%)		
	
	Meghalaya	Assam	Total	Meghalaya	Assam	Total
*bla*_TEM_	15 (2.79)	0.00	15 (1.73)	0.00	0.00	0.00
*bla*_CTX-M_	3 (0.55)	6 (1.01)	9 (1.03)	0.00	0.00	0.00
*bla*_CMY_	21 (3.91)	3 (0.90)	24 (2.76)	3 (12.50)	0.00	3 (10.00)
*bla*_TEM_ and *bla*_CTX-M_	0.00	0.00	0.00	0.00	0.00	0.00
*bla*_TEM_ and *bla*_CMY_	15 (2.79)	0.00	15 (1.73)	3 (12.50)	3 (50.00)	6 (20.00)
*bla*_CTX-M_ and *bla*_CMY_	0.00	3 (0.90)	3 (0.34)	0.00	0.00	0.00
*bla*_TEM_ *bla*_CTX-M_ and *bla*_CMY_	0.00	0.00	0.00	3 (12.50)	0.00	3 (10.00)

Figures in parenthesis are percentile values

### In vitro HGT

**Figure-3:**
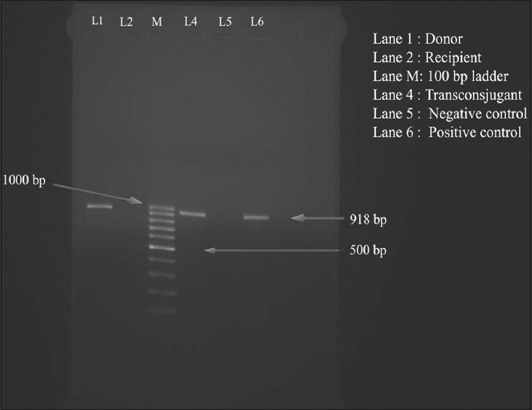
Polymerase chain reaction assay for detection of (*bla*_TEM_) gene (918 bp) in transconjugant for confirmation of horizontal gene transfer conjugation. Lane 1: Donor; Lane 2: Recipient; Lane M: 100 bp DNA ladder; Lane 4: Transconjugant; Lane 5: Negative control; and Lane 6: Positive control.

Transconjugants were detected only in broth mating and filter paper mating methods. The isolated transconjugants showed identical resistance profiles and all contained in a large plasmid. Only *bla*_TEM_ gene could be transferred from the donor to recipient in the absence of antibiotic pressure. The target gene was detected from the transconjugants obtained from the plate, whereas it was absent in recipient strain, as detected by PCR assay (Figure-3). The resistance plasmid transfer rate from donor to the recipient was 5.5×10^−5^ in filter paper mating and 5.7×10^−5^ by broth mating in respect to the recipient.

### *In vivo* HGT

Transconjugants were obtained from both the experimental groups and were resistant to streptomycin and carrying plasmids with *bla*_TEM_ gene (Figure-3). The frequency of transconjugants from antibiotic treatment group (8.8×10^−4^) was higher than the non-antibiotic group (6.5×10^−5^). Transconjugants were detected from the mouse with antibiotic treatment group from the 4^th^-day postinoculation, whereas, it could be detected from the 6^th^-day postinoculation from the group without any antibiotic pressure.

## Discussion

The present study revealed that the prevalence of *bla*_CMY_ gene in *E. coli* was highest in both the states compared to the other two genes. *bla*_CMY_ is the member of the plasmid-mediated AmpC like enzyme, which has been shown to be the most common gene associated with ceftriaxone resistance among *E. coli* and *Salmonella* serovars in food animals and humans [9]. Detection of *bla*_CMY_ in pigs also reported by Mandakini [8] from India in feces of animals, particularly pigs by various workers abroad with nearly similar kind of result [10].

*bla*_TEM_ gene was only recorded in *E. coli* from Meghalaya, whereas all the *E. coli* isolates from Assam were recorded as negative. Similarly, *Salmonella* isolates from Meghalaya and Assam were recorded as positive for *bla*_TEM_ gene. Lalzampuia *et al*. [11] reported from the same laboratory that none of the 102 *E. coli* isolates from pigs in Mizoram were carrying *bla*_TEM_ gene. The *bla*_TEM_ genes also have a tendency to mutate and secrete enzymes with extended spectrum of activity, which could have accounted for the high resistance to ampicillin in the population studied [12].

Similarly, the population of *bla*_CTX-M_ gene positive in *E. coli* was higher in Assam compared to Meghalaya. In contrast to that *Salmonella* isolates carrying *bla*_CTX-M_ gene from both the states were nearly similar. In comparison to *bla*_TEM_ and *bla*_CMY_ genes, the prevalence of *bla*_CTX-M_ gene in both *E. coli* and *Salmonella* from Meghalaya and Assam was found to be lower. Our result is also in corroboration with the report of other workers [13-15].

In this study, DS-54 was successfully cured using acridine orange at a concentration of 2-2.5 mg/ml. Akortha and Filgona [16] used 0.10 mg/ml of acridine orange for plasmid curing of *E. coli* isolates. Although curing provides only the preliminary evidence that genetic traits are of extrachromosomal nature, the loss of growth on antibiotic containing plates shows that the multidrug resistance genes may be plasmid-borne. It is, however, important to note that all antibiotic resistance genes are plasmid mediated [17]. Sometimes copies of the plasmids lying closer to the membrane are eliminated by curing agents, while those lying closer to the nucleus may escape the curing effect [18].

During the HGT study, the plasmids carrying *bla*_TEM_ gene could be transferred horizontally to the recipient isolates only by broth mating and filter paper mating. The resistance gene was not transferred in plate mating. The resistance plasmid transfer rate from donor to the recipient by broth mating was 5.7×10^−5^/recipient, whereas in filter plate mating it was 5.5×10^−5^/recipient. Faure *et al*. [15] reported the mean frequency of transfer of resistance plasmid from *Salmonella* to *E. coli* at 5.9 to 5.7×10^−8^/donor. The frequency of the transfer of resistance plasmids from donor to the recipient may vary depending on the copy number of the conjugative plasmids in the donor strain.

Similarly, in *in vivo* experiment using mice model the frequency of transfer of conjugative plasmid carrying antibiotic resistance genes increased in the presence of antibiotic selection pressure. There was negligible difference in the frequencies of transfer of the plasmids from donor to recipient (5.7×10^−5^ vs^.^ 6.5×10^−5^/recipient. However, the frequency of transfer of the target plasmid was significantly increased (8.8×10^−4^) in the presence of antibiotic selection pressure in mice model. Similar kinds of the report are also published by several workers from abroad [14,15]. *In vitro* experiment showed the transfer of the plasmids ranging from 108 to 157 kb, while *in vivo* conjugation experiment showed a transfer of smaller sized plasmids [19].

In this study, only *bla*_TEM_ gene could be transferred from the donor to recipient strain both by *in vivo* method of HGT. Transfer of *bla*_TEM_ gene carrying plasmid from donor to recipient in the presence of streptomycin is also reported by Chen *et al*. [20]. *bla*_TEM_ is associated with plasmid-mediated beta-lactamase, which confers resistance to the third generation of cephalosporins, particularly ceftazidime, and cefotaxime [21]. *bla*_TEM_ beta-lactamases are maintained the ability of the bacteria to hydrolyze third-generation cephalosporins but also demonstrate an inhibitor resistance [21].

## Conclusion

This study showed that the ESBL producing genes (*bla*_TEM_, *bla*_CTX-M_, and *bla*_CMY_) are prevalent in the enteric bacteria of pig population in Meghalaya and Assam. Presence of such genes in extrachromosomal component of enteric bacteria indicates their potential to transfer within species or inter-species in animal population in this region. It also needs to understand the different methods of the possible spread of resistant bacterial population in the society to curb the menace of further development of multidrug-resistant bacteria in the environment.

## Authors’ Contributions

AL: Collection of samples, processing for isolation and identification of bacteria, *in vitro* and *in vivo* HGT assay. TKD: Genesis of concept of the work and preparation of the manuscript. PR: Detection of ESBLs genes by PCR assays. PKS: AMR sensitivity assay and data analysis. All authors read and approved the final manuscript.
